# Supplementary material for: Short-Term Audiovisual Spatial Training Enhances Electrophysiological Correlates of Auditory Selective Spatial Attention
Source: Front Neurosci. 2021 Jul 1;15:645702. doi: 10.3389/fnins.2021.645702 (PMC8280319; doi:10.3389/fnins.2021.645702)
Supplement: Supplementary file 1 [file Image_1.pdf]

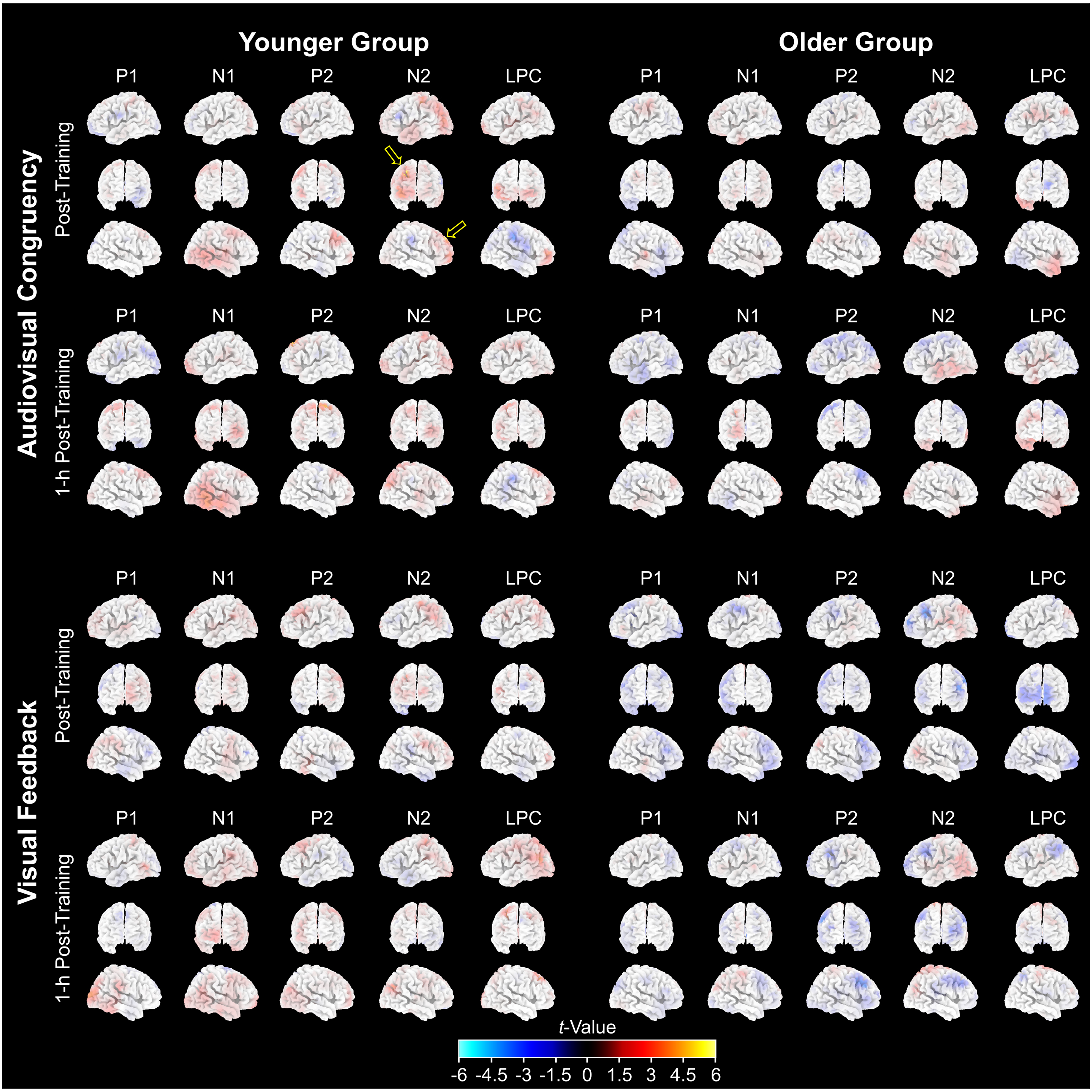

Supplementary Figure 1. Contrast of training vs. control conditions, as revealed by sLORETA. Statistical comparisons were conducted with baseline-normalized data for all training conditions (audiovisual-congruency training, visual-feedback training), blocks (post-training, 1-h post-training), and main ERP components (P1, N1, P2, N2, LPC). Data were mapped onto a standard 3-D brain template (Colin of sLORETA; left, frontal, and right view). Color coding shows *t*-values, with warm colors indicating increase of electrical activity and cold colors indicating decrease of electrical activity after training intervention. Significant peak activity ($p < 0.05$; arrows) was found exclusively at the time of the N2 in the younger group immediately after audiovisual-congruency training.
